# On a Robust MaxEnt Process Regression Model with Sample-Selection

**DOI:** 10.3390/e20040262

**Published:** 2018-04-09

**Authors:** Hea-Jung Kim, Mihyang Bae, Daehwa Jin

**Affiliations:** Department of Statistics, Dongguk University-Seoul, Pil-Dong 3Ga, Chung-Gu, Seoul 100-715, Korea

**Keywords:** Gaussian process model, hierarchical Bayesian methodology, robust sample-selection MaxEnt process regression model, Markov chain Monte Carlo, sample-selection bias, bias correction, 62G08, 62F15

## Abstract

In a regression analysis, a sample-selection bias arises when a dependent variable is partially observed as a result of the sample selection. This study introduces a Maximum Entropy (MaxEnt) process regression model that assumes a MaxEnt prior distribution for its nonparametric regression function and finds that the MaxEnt process regression model includes the well-known Gaussian process regression (GPR) model as a special case. Then, this special MaxEnt process regression model, i.e., the GPR model, is generalized to obtain a robust sample-selection Gaussian process regression (RSGPR) model that deals with non-normal data in the sample selection. Various properties of the RSGPR model are established, including the stochastic representation, distributional hierarchy, and magnitude of the sample-selection bias. These properties are used in the paper to develop a hierarchical Bayesian methodology to estimate the model. This involves a simple and computationally feasible Markov chain Monte Carlo algorithm that avoids analytical or numerical derivatives of the log-likelihood function of the model. The performance of the RSGPR model in terms of the sample-selection bias correction, robustness to non-normality, and prediction, is demonstrated through results in simulations that attest to its good finite-sample performance.

## 1. Introduction

The Bayesian nonparametric method is a powerful approach for regression problems when the shape of the underlying regression function is unknown, the function may be difficult to evaluate analytically, or other requirements such as design costs may complicate the process of information acquisition process. Bayesian orthogonal basis expansion regression, spline smoothing regression, wavelet regression, and Gaussian process regression (GPR) are powerful nonparametric Bayesian approaches that address these regression problems. These regression techniques have been extensively used in fields such as psychology, data science, engineering, neuroscience, and fishery [1,2,3,4,5].

Sample selection (or incidental truncation) in regression analysis is known to often arise in a wide variety of practical problems and standard analysis of data with sample selection leads to biased results because the selected sample represents only a subset of a full population; see [6,7,8]. Regression analysis also has problems regarding sensitivity to outliers and departures from the normality of the dependent variable (see [9,10]). Thus, when one implements nonparametric Bayesian regression with non-normal data with sample selection, the selection mechanism and non-normality of the data must be jointly modeled with the Bayesian nonparametric regression model to correct the sample-selection bias and to implement a robust statistical inference. In this regard, several estimation procedures have been considered in the literature to produce robust linear regression models that are subject to sample selection including for instance [6,7], for frequentist methods, and [8,9,11] for Bayesian methods. See [9,12] to obtain robust Bayesian sample-selection models other than the regression model. In addition, no studies have generalized a nonparametric regression model to deal with non-normal data with the sample selection.

The objective of this paper is to introduce the Maximum Entropy (MaxEnt) process regression model as a new Bayesian nonparametric regression model and to then generalize this model to propose a robust sample-selection Bayesian nonparametric regression model along with its inferential methodology. The MaxEnt process regression model is obtained by assuming a MaxEnt prior distribution for its nonparametric regression function, and it includes the GPR model as a special case. This provides a relationship between the MaxEnt nonparametric regression approach and the rationale to conduct a Gaussian regression analysis. This study focuses on the GPR model as a special MaxEnt process regression model and a powerful analysis model towards nonparametric regression problems. Then, the GPR model is generalized to obtain a robust sample-selection Gaussian process regression (RSGPR) model. This RSGPR model extends the GPR model to account for the sample selection scheme, and it is robust when the data are heavy-tailed or contain outliers.

The RSGPR model consists of two components. The first is a robust GPR model that determines the level of the dependent variable of interest and the second is an equation that describes the selection mechanism that determines whether we have observed the dependent variable or not. The sample-selection bias arises when these two components are correlated and must be modeled jointly. A Bayesian hierarchical methodology is developed here to estimate the RSGPR model. This methodology relies on a stochastic representation technique (see, e.g., [13]) to set up the Bayesian hierarchy of the RSGPR model, and it has three attractive features. First, given the likelihood function of the model, the posterior of its parameters does not belong to any well-known parametric family, but the methodology uses a simple Markov chain Monte Carlo (MCMC) algorithm that does not resort to generating random draws from the complex posterior. Second, the output of the algorithm not only provides a Bayesian analogue of confidence intervals for the regression function, but it also readily gives an indication of the presence (or absence) of the sample-selection bias. Third, if there is prior information, such as restrictions on the regression function, such information can be incorporated easily through a prior distribution.

The remainder of this study is organized as follows. Section 2 introduces the MaxEnt process regression model that strictly includes the GPR model. Then, this section formulates the RSGPR model that is obtained by incorporating the GPR model with a class of scale mixtures of normal errors as well as a selection model comprising a class of scale mixtures of the probit sample selection equations. Properties of this RSGPR model are studied including the exact distribution of a selected observation, a stochastic representation, a distributional hierarchy, and the magnitude of the sample-selection bias. In Section 3, we construct a Bayesian hierarchical model for inference in the RSGPR model by exploiting the stochastic representation and distributional hierarchy. Then we develop a Bayesian estimation methodology based on the hierarchical model to provide a simple estimation procedure for the RSGPR model. We further construct a computationally feasible MCMC algorithm through a Bayesian hierarchical approach. Section 4 examines the finite-sample performance of the method through a limited but informative simulation. This numerical illustration shows the usefulness of the RSGPR model for the Gaussian process regression analysis of non-normal data with the sample selection. The study then concludes with a discussion in Section 5. Proofs and additional details are provided in the Appendix A.

## 2. Robust Sample-Selection GPR Model

### 2.1. MaxEnt Process Regression Model

Consider the following nonparametric regression model,

(1) $$y_{n}=\eta_{n}\left(x\right)+\epsilon_{n},$$

where $y_{n}={\left(y_{1},\ldots ,y_{n}\right)}^{⊤}$ is n×1 vector of responses, $y_{i}=\eta \left(x_{i}\right)+\epsilon_{i},$ $\eta_{n}\left(x\right)={\left(\eta \left(x_{1}\right),\ldots ,\eta \left(x_{n}\right)\right)}^{⊤}$ is n×1 vector of regression function values satisfying $\eta \left(x_{i}\right)=E\left[y_{i}|x_{i}\right],$ i=1,…,n, $x={\left(x_{1},\ldots ,x_{n}\right)}^{⊤}$ is the n×p design matrix, and $\epsilon_{n}={\left(\epsilon_{1},\ldots ,\epsilon_{n}\right)}^{⊤}$ is a n×1 vector of i.i.d. random noises with zero mean vector. In the basic model structure of (1), the parametric form of the regression function $\eta_{n}\left(x\right)$ is not assumed, but $\eta_{n}\left(x\right)$ is assumed to have specific types of functional structure. For example, $\eta_{n}\left(x\right)$ can be represented with a Fourier series [14], splines [15], kernels [16] and others.

In the Bayesian nonparametric regression, we assume that the regression function (or signal term) $\eta_{n}\left(x\right)$ is a random function that follows a particular distribution. This distribution is subjective in the sense that the distribution reflects our uncertain prior information regarding the function. Sometimes, we have a situation in which partial prior information on $\eta_{n}\left(x\right)$ is available, outside of which it is desirable to use a prior that is as non-informative as possible. In this situation, Boltzmann’s maximum entropy theorem (see, e.g., [17]) yields a maximum entropy prior $\pi_{max}\left(\eta_{n}\left(x\right)\right)$ that is an exponential form and maximizes the entropy,

$$H\left(\pi \right)=-\int_{R^{n}}\pi \left(\eta_{n}\left(x\right)\right)\log\pi \left(\eta_{n}\left(x\right)\right)d\eta_{n}\left(x\right),$$

in the presence of partial information for various moment functions of $\eta_{n}\left(x\right).$ In a special case where we only have partial prior information about the mean vector and covariance matrix functions of $\eta_{n}\left(x\right)$ of the Bayesian nonparametric regression model (1), Boltzmann’s maximum entropy theorem yields the following prior distribution of $\eta_{n}\left(x\right)$.

**Lemma** **1.** *Let n×1 regression function vector $\eta_{n}\left(x\right)$ have a prior distribution on $R^{n}$ whose partial information on the mean and covariance functions are $m\left(x\right)={\left(m\left(x_{1}\right),\ldots ,m\left(x_{n}\right)\right)}^{⊤}$ and $K\left(x\right)=\{\kappa \left(x_{i},x_{j}\right)\},$ respectively. Then the maximum entropy prior of $\eta_{n}\left(x\right)$ is*

(2) $$\pi_{max}\left(\eta_{n}\left(x\right)\right)={\left(2\pi \right)}^{-n/2}{\left|K\left(x\right)\right|}^{-1/2}\exp\{-\frac{1}{2}{\left(\eta_{n}\left(x\right)-m\left(x\right)\right)}^{⊤}K{\left(x\right)}^{-1}\left(\eta_{n}\left(x\right)-m\left(x\right)\right)\}$$

*for $\eta_{n}\left(x\right)\in R^{n}.$ This is a density of GP(m(x),K(x)), a Gaussian process defined by the mean function m(x) and the covariance function K(x).*

Note that the Gaussian process $GP\left(m(x),K(x)\right)$ defines a collection of random functions wherein any finite subset of the process has multivariate normal (Gaussian) distribution. From now on, we will write the Gaussian process as $\eta_{n}\left(x\right)∼GP\left(m(x),K(x)\right).$ The only restriction on the Gaussian process is that the covariance function K(x) must be an n×n positive definite symmetric (pds) matrix. If K(x) is not a pds matrix, then the corresponding value of $H\left(\pi_{max}\right)=p\left(1+\log\left(2\pi \right)\right)/2+\log(|K\left(x\right)\left|\right)/2$ will not be defined (see, e.g., [18]). As a result, this paper introduces yet another Bayesian nonparametric regression model by combining the regression model (1) and the MaxEnt prior in Lemma 1 and introducing a normal regression error distribution. The model is named as a “MaxEnt process regression model” and defined by

(3) $$\begin{array}{ccc} y_{n} & = & \eta_{n}\left(x\right)+\epsilon_{n},\;\epsilon_{n}∼N_{n}\left(0,\sigma^{2}I_{n}\right),\\ \sigma^{2} & ∼ & IG(\nu_{1},\nu_{2}),\\ \eta_{n}\left(x\right) & ∼ & GP\left(m(x),K(x)\right), \end{array}$$

where $N_{n}\left(0,\sigma^{2}I_{n}\right)$ is an *n*-variate normal distribution with mean vector 0 and covariance matrix $\sigma^{2}I_{n},$ $IG(\nu_{1},\nu_{2})$ denotes an inverse gamma distribution with shape parameter $\nu_{1}$ and scale parameter $\nu_{2},$ $\eta_{n}\left(x\right)$ is independent of $\sigma^{2}$ and $\epsilon_{n},$ the mean function $m(x_{i})$ reflects the expected function value at input $x_{i}$, i.e., $m\left(x_{i}\right)=E\left[\eta \left(x_{i}\right)\right],$ and the covariance function $\kappa (x_{i},x_{j})$ models the dependence between the function values at different input points $x_{i}$ and $x_{j},$ i.e.,$\kappa \left(x_{i},x_{j}\right)=E\left[\left(\eta \left(x_{i}\right)-m\left(x_{i}\right)\right)\left(\eta \left(x_{j}\right)-m\left(x_{j}\right)\right)\right],$ i,j=1,…,n. See [19] for choice of an appropriate covariance function based on assumptions such as smoothness and likely patterns to be expected in the data. A commonly used isotropic covariance function in practice is the squared exponential covariance function given by

(4) $$\kappa \left(x_{i},x_{j}\right)=Cov\left(\eta \left(x_{i}\right),\eta \left(x_{j}\right)\right)=u_{0}\exp\left\{-\frac{w_{0}}{2}{\left(x_{i}-x_{j}\right)}^{⊤}\left(x_{i}-x_{j}\right)\right\},$$

where $u_{0}$ and $w_{0}$ are hyperparameters and which are relevant for the shape of MaxEnt process regression. Here $u_{0}$ stands for global scale of the covariance matrix K(x) and $w_{0}$ stands for smoothing parameter, respectively.

We can easily see that the MaxEnt process regression model (5) is the same as the GPR model considered by [19,20]. This proves the following corollary and can hence be used as an information theoretic justification for using the GPR model as a Bayesian approach for a nonparametric regression analysis.

**Corollary** **1.** Suppose $E[\eta_{n}\left(x\right)]=m\left(x\right)$ and $\mathrm{Cov}(\eta_{n}\left(x\right))=K\left(x\right)$ are all the prior information on $\eta_{n}\left(x\right)$ for the Bayesian nonparametric regression model (1). Then MaxEnt prior distribution of $\eta_{n}\left(x\right)$ is $GP\left(m(x),K(x)\right)$ which defines the GPR model.

According to Corollary 1, we shall denote the MaxEnt process regression model by the GPR model. When there is no functional constraint in the GPR model, then the prior specification in the model (3) can be used, and posterior inference can be performed without difficulty. It is seen, from [20], that the conditional posterior distribution of $\eta_{n}\left(x\right)$ is normal with the mean and covariance given by

(5) $$\begin{array}{ccc} E[\eta_{n}\left(x\right)|\left(y_{n},x\right),\sigma^{2}] & = & K\left(x\right){\left(K\left(x\right)+\sigma^{2}I_{n}\right)}^{-1}y_{n}+\sigma^{2}I_{n}{\left(K\left(x\right)+\sigma^{2}I_{n}\right)}^{-1}m\left(x\right),\\ \mathrm{Var}[\eta_{n}\left(x\right)|\left(y_{n},x\right),\sigma^{2}] & = & {\left(K{\left(x\right)}^{-1}+\sigma^{-2}I_{n}\right)}^{-1}=\sigma^{2}K\left(x\right){\left(K\left(x\right)+\sigma^{2}I_{n}\right)}^{-1}. \end{array}$$

However, in the GPR analysis, a sample-selection scheme often applies to the response variable that results in missing not at random (MNAR) observations on the variable. In this case, the regression analysis using only the selected cases will lead to biased results (see, e.g., [6,7,8]). This study provides a bias correction procedure for the GPR analysis with MNAR data generated from the sample-selection scheme. For the analysis, we propose a robust sample-selection GPR (RSGPR) model based on a family of scale mixtures of normal (SMN) distributions (see [21,22] for details). This approach reflects the MNAR mechanism as well as its robustness against departures from the normality assumption (see, e.g., [11,12]), and proposes a robust GPR model to analyze the partially observed sample-selection data.

### 2.2. Proposed Model

We propose the RSGPR model through the following steps. First, we modify the GPR model (3) by incorporating the SMN error distribution for a robust GPR analysis. Then, we connect the robust GPR model directly to a sample-selection model by introducing some latent variables to explain the partially observed sample-selection data. To model the sample-selection mechanism, we need to introduce some notation for the partially observed data.

Let $s_{i}$ be a binary variable that takes on value 1 if $y_{i}$ of subject *i* is observed using the sample-selection scheme, and 0 if that of the subject is not observed using the same scheme. Then, we introduce the following RSGPR model to represent the regression equation of the observable variable $y_{i}:$

(6) $$\begin{array}{ccc} y_{i} & = & \left\{\begin{array}{cc} \eta \left(x_{i}\right)+\epsilon_{i}\;\;\; & \mathrm{for}\;\;s_{i}=I\left(z_{i}\geq 0\right),\\ \mathrm{missing}\;\;\; & \mathrm{for}\;\;s_{i}=I\left(z_{i}<0\right), \end{array}\right.\\ z_{i} & = & v_{i}^{⊤}\gamma +\varepsilon_{i},\;\;i=1,\ldots ,n,\;\;\left(\begin{array}{c} \epsilon_{i}\\ \varepsilon_{i} \end{array}\right)\overset{iid}{∼}{SMN}_{2}\left(0,\Sigma ,\delta ,G\right),\\ \eta_{n}\left(x\right) & ∼ & GP\left(m(x),K(x)\right), \end{array}$$

where I(·) is an indicator function, ${\mathrm{SMN}}_{2}\left(0,\Sigma ,\delta ,G\right),$ a scale mixture of bivariate normal distributions with mixture function δ(ω) and mixing variable ω∼G. Here $\gamma ={\left(\gamma_{1},\ldots ,\gamma_{q}\right)}^{⊤}$ and $\Sigma =\left(\begin{array}{cc} \sigma^{2} & \rho \sigma \\ \rho \sigma & 1 \end{array}\right)$ are parameters to be elicited by using the priors $p_{0}\left(\gamma \right)$ and $p_{0}\left(\Sigma \right)$.

Without loss of generality, we assume that the single sample selection scheme $s_{i}=I\left(z_{i}\geq 0\right)$ is applied to a random sample of *n* observations ($y_{i}$’s) associated with the model (1) and gives only the first $n_{1}$ observed values of $y_{i}$’s out of the *n*$\left(n>n_{1}\right)$ observations according to the sample-selection scheme. Thus, the overall available data information of the RSGPR model consists of the set of $s_{i}$ binary values and the $n_{1}$-tuples of observations $\left(y_{i},v_{i}\right)$ corresponding to individuals with $s_{i}=1,$ while $v_{i}$ for those with $s_{i}=0.$ The purpose of this study is to estimate the regression function $\eta_{n}\left(x\right)$ based on partially observed data (i.e., sample-selection data) with size $n_{1}.$

For fixed $\eta (x_{i})$, the density of the RSGPR model (6) is composed of a continuous component $h(y_{i}|s_{i}=1)$ and a discrete component $p(s_{i}).$ The discrete component is

(7) $$\begin{array}{ccc} p(s_{i}) & = & {\left[\bar{F}\left(C_{i};0,1\right)\right]}^{s_{i}}{\left[1-\bar{F}\left(C_{i};0,1\right)\right]}^{1-s_{i}}, \end{array}$$

where $\bar{F}\left(C_{i};d,\tau \right)=\int_{0}^{\infty }\bar{\Phi }\left(C_{i};d,\delta \left(\omega \right)\tau \right)dG\left(\omega \right)$ with a selection interval $C_{i}=\left(\alpha_{i},\infty \right),$ $\alpha_{i}=-v_{i}^{⊤}\gamma ,$ and $\bar{\Phi }\left(C_{i};d,\kappa \left(\omega \right)\tau \right)=\int_{C_{i}}\phi \left(x;d,\delta \left(\omega \right)\tau \right)dx$ denotes the probability of the interval $C_{i}$ under the N(d,δ(ω)τ) distribution with the density ϕ(x;d,δ(ω)τ). The continuous component is a density of $\left[y_{i}|\eta \left(x_{i}\right),s_{i}=1\right]\overset{d}{=}\left[y_{i}|\eta \left(x_{i}\right),\;\varepsilon_{i}\in C_{i}\right]$ for $i=1,\ldots ,n_{1}.$ This density is given by

(8) $$\begin{array}{ccc} h(y_{i}|s_{i}=1) & = & \frac{\int_{0}^{\infty }\phi \left(y_{i};\;\eta \left(x_{i}\right),\delta \left(\omega \right)\sigma^{2}\right)\bar{\Phi }\left(C_{i};\theta_{\varepsilon_{i}|y_{i}},\delta \left(\omega \right)\sigma_{\varepsilon_{i}|y_{i}}^{2}\right)dG\left(\omega \right)}{\bar{F}\left(C_{i};0,1\right)},\;\;y_{i}\in R, \end{array}$$

where $\theta_{\varepsilon_{i}|y_{i}}=\rho \left[y_{i}-\eta \left(x_{i}\right)\right]/\sigma ,$ and $\sigma_{\varepsilon_{i}|y_{i}}^{2}=1-\rho^{2}.$ This distribution is essentially a member of the class of skew-scale mixtures of normal (skew-SMN) distributions discussed by [13,23,24]. We will denote the distribution law of $\left[y_{i}|\eta \left(x_{i}\right),s_{i}=1\right]$ with density (8) by skew-$\mathrm{SMN}(C_{i};\theta_{i},\Sigma ,\delta ,G),$ where $\theta_{i}={\left(\eta \left(x_{i}\right),0\right)}^{⊤}.$ The following lemma is useful to generate the partially observed $y_{i}$’s and indicate the difference between the RSGPR model (6) and the GPR model (3).

**Lemma** **2.** *For a given value of $\eta (x_{i}),$ the selected observation $\left[y_{i}|\eta \left(x_{i}\right),s_{i}=1\right]$ for the RSGPR model can be represented by the following two-stages of distributional hierarchy:*

(9) $$\begin{array}{ccc} {}[y_{i}|\omega ,\eta \left(x_{i}\right),s_{i}=1]\; & \overset{d}{=} & \;\eta \left(x_{i}\right)+\rho \sigma Z_{C_{i}}+\sigma {\left(1-\rho^{2}\right)}^{1/2}U_{i},\;\;i=1,\ldots ,n_{1},\\ \omega & ∼ & G(\omega ), \end{array}$$

*where $U_{i}\overset{iid}{∼}N\left(0,\delta \left(\omega \right)\right)$ and $Z_{C_{i}}\overset{ind}{∼}{\mathrm{TN}}_{C_{i}}\left(0,\delta \left(\omega \right)\right)$ are independent conditionally on ω, and ${\mathrm{TN}}_{C_{i}}\left(0,\delta \left(\omega \right)\right)$ denotes a N(0,δ(ω)) distribution truncated to the interval $C_{i}=\left(\alpha_{i},\infty \right).$*

Lemma 2 shows that the RSGPR model applies to relax the classic assumption of the underlying normality as well as to reflect the sample-selection scheme. This lemma also indicates that the partially observed data $y_{i}$’s does not represent a random sample from the GPR model generating $y_{i}$’s, even after controlling for the regression function $\eta (x_{i}).$ If we want to apply a GPR analysis to the partially observed sample-selection data, a fitted model should be the RSGPR model. The RSGPR model changes depending on the choice of the distribution of ω and its function δ(ω). In the special case wherein the distribution of ω degenerates at δ(ω)=1, the RSGPR model produces a sample-selection Gaussian process normal error regression (SGPR${}_{N}$) model. When we choose ω∼G(ν/2,ν/2), a gamma distribution with mean 1 and $\delta \left(\omega_{i}\right)=1/\omega_{i},$ the model becomes a sample-selection Gaussian process $t_{\nu }$ error regression (SGPR${}_{t_{\nu }}$) model, allowing to regulate the tail distribution of the model by means of the degrees of freedom. We also see that the RSGPR model strictly includes the GPR model because the latter is obtained by setting ρ=0. For the remainder of this study, we use the symbols in the preceding sections with the same definitions.

### 2.3. The Sample-Selection Bias

As indicated by the density (8) and Lemma 2 the selected observations $\left[y_{i}|s_{i}=1\right]$’s do not represent a random sample from the GPR model generating $y_{i}$’s, but they are missing not at random (MNAR) [25] inducing a sample-selection bias. The following results on the sample-selection bias are noted in the Bayesian estimation of the GPR model with the partially observed data.

**Lemma** **3.** *Given the RSGPR model (6), a stochastic representation of conditional posterior distribution of the regression function $\eta_{n_{1}}={\left(\eta \left(x_{1}\right),\ldots ,\eta \left(x_{n_{1}}\right)\right)}^{⊤}$ is*

(10) $$\begin{array}{ccc} \left[\eta_{n_{1}}|y,\omega ,\Psi \right] & \overset{d}{=} & \theta_{1}+\Gamma \Omega_{2}^{-1}W_{1}^{C_{\beta }}+W_{2}, \end{array}$$

*where $\Psi =\{\sigma^{2},\rho ,\gamma \},$$W_{1}∼N_{n_{1}}\left(0,\Omega_{2}\right)$ and $W_{2}∼N_{n_{1}}\left(0,\Omega_{1}-\Gamma \Omega_{2}^{-1}\Gamma^{⊤}\right)$ are independent random vectors, $W_{1}^{C_{\beta }}\overset{d}{=}\left[W_{1}|W_{1}\in C_{\beta }\right],$$C_{\beta }=\cap_{i=1}^{n_{1}}\left\{w_{1i};\;\beta_{i}\leq w_{1i}\leq \infty \right\},$$W_{1}={\left(w_{11},\ldots ,w_{1n_{1}}\right)}^{⊤},$$\beta_{i}=\left(\alpha_{i}-\theta_{2i}\right)/\sqrt{\delta (\omega )},$$\theta_{1}=K_{11}{\left(x\right)}^{-1}H^{-1}y_{n_{1}}+\delta \left(\omega \right)\sigma^{2}H^{-1}m_{1}\left(x\right),$$\theta_{2}={\left(\theta_{21},\ldots ,\theta_{2n_{1}}\right)}^{⊤}=\rho \left(y_{n_{1}}-m_{1}\left(x\right)\right)/\sigma ,$$\Gamma =-\rho \Omega_{1}/\sigma ,$$H=\left(K_{11}\left(x\right)+\delta \left(\omega \right)\sigma^{2}I_{n_{1}}\right),$$\Omega_{1}=\delta \left(\omega \right)\sigma^{2}K_{11}\left(x\right)H^{-1},\;$$\Omega_{2}=\left(1-\rho^{2}\right)I_{n_{1}}+\rho^{2}\Omega_{1}/\sigma^{2},\;$$y_{n_{1}}={\left(y_{1},\ldots ,y_{n_{1}}\right)}^{⊤}$ be an $n_{1}\times 1$ observed vector, $m_{1}\left(x\right)={\left(m\left(x_{1}\right),\ldots ,m\left(x_{n_{1}}\right)\right)}^{⊤},$ and $K_{11}\left(x\right)$ is the first $n_{1}\times n_{1}$ diagonal sub-matrix of K(x).*

As shown in Lemma 3, if we use the partially observed $y_{i}$’s to estimate the GPR model, the existence of the truncated normal distribution term (i.e., $W_{1}^{C_{\beta }}$) in Equation (10) induces a biased estimation of the regression function. Note that the distribution becomes normal (i.e., $W_{1}∼N_{n_{1}}\left(0,\Omega_{2}\right)$) in the GPR model for the case where $y_{i}$’s are fully observed. Therefore, the usual estimation of the regression function based on the GPR model will produce inconsistent results when ρ≠0. This clearly reveals that sample-selection bias occurs in Bayes estimation of the regression function $\eta_{n_{1}}.$ The magnitude of this bias is as follows.

**Corollary** **2.** *Instead of the SGPR${}_{N},$ if the GPR model is used for estimating $\eta_{n_{1}}$ based on observed data $y_{n_{1}}$ then a sample-selection bias occurs in its conditional posterior mean. This bias is*

$$E\left[\eta_{n_{1}}|y_{n_{1}},\Psi \right]-E\left[\eta_{n_{1}}|y_{n_{1}},\sigma^{2}\right]=-{\left(\frac{\rho }{\sigma }I_{n_{1}}+\frac{\sigma (1-\rho^{2})}{\rho }\Omega_{1}^{*-1}\right)}^{-1}\xi ,$$

where $\xi =E\left[W_{1}^{C_{\beta }}\right]={\left(\xi_{1},\ldots ,\xi_{n_{1}}\right)}^{⊤},$$\xi_{i}=\omega_{ii}^{*}\phi \left(\beta_{i};0,\omega_{ii}^{*}\right)/\left[1-\Phi \left(\beta_{i}/\sqrt{\omega_{ii}^{*}}\right)\right],$$\omega_{ii}^{*}$ denotes the i-th diagonal element of $\Omega_{2}^{*},$$\Omega_{1}^{*}=\Omega_{1}{|}_{\delta (\omega )=1},$ and $\Omega_{2}^{*}=\Omega_{2}{|}_{\delta (\omega )=1}.$

The sample-selection bias in calculating the marginal effect (or propensity) of a predictor can be also expected.

**Corollary** **3.** *Suppose that $v_{ki}=x_{ki},$ where $v_{ki}$ and $x_{ki}$ are k-th element of $v_{i}$ and $x_{i},$ then difference in the marginal effect of the predictor $x_{ki}$ on the selected observation $y_{i}$ between the RSGPR model and the GPR model is*

(11) $$\begin{array}{c} \gamma_{k}\rho \sigma E_{\omega }\left[\frac{1}{\delta (\omega )}\left(\delta_{2}\left(v_{i}^{⊤}\gamma ,\omega \right)-\delta_{1}{\left(v_{i}^{⊤}\gamma ,\omega \right)}^{2}\right)\right], \end{array}$$

*where $\gamma_{k}$ is the k-th element of γ,*

$$\begin{array}{ccc} b_{1}\left(v_{i}^{⊤}\gamma ,\omega \right) & = & \delta \left(\omega \right)\phi \left(\alpha_{i};0,\delta \left(\omega \right)\right)/\left[1-\Phi \left(\alpha_{i}/\sqrt{\delta (\omega )}\right)\right],\\ b_{2}\left(v_{i}^{⊤}\gamma ,\omega \right) & = & \alpha_{i}\delta \left(\omega \right)\phi \left(\alpha_{i};0,\delta \left(\omega \right)\right)/\left[1-\Phi \left(\alpha_{i}/\sqrt{\delta (\omega )}\right)\right], \end{array}$$

*$\alpha_{i}=-v_{i}^{⊤}\gamma ,$ and $E_{\omega }$ denotes the expectation is taken with respect to the distribution of ω∼G(ω).*

**Figure 1:**
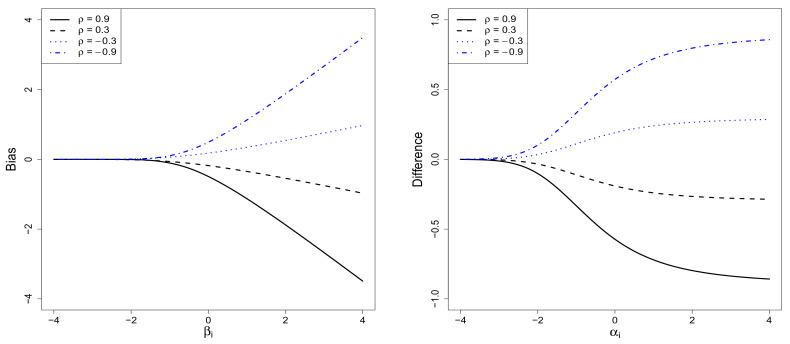
Graphs of the sample-selection bias and the difference in marginal effect of the *k*-th predictor.

To compare the SGPR${}_{N}$ model with the GPR model, various values of the sample-selection bias associated with the posterior mean (see, Corollary 2) and the difference in the marginal effect of the *k*-th predictor (see, Corollary 3) were calculated and are depicted in Figure 1. For the calculation, we set σ=1, $\gamma_{k}=1,$ and $K_{11}\left(x\right)=0.5I_{n_{1}}+0.51_{n_{1}}1_{n_{1}}^{⊤}/n_{1},$ an intra-class covariance matrix, where $1_{n_{1}}$ is an $n_{1}\times 1$ summing vector whose elements are all one. The left panel in Figure 1 is a graph of the sample-selection bias for different values of $\beta_{i}$ and ρ. This graph shows the values of the first element of the bias vector given in Corollary 2. From the graph, we see that the sample-selection bias occurs in the GPR analysis with sample selection, and its magnitude becomes larger as the values of |ρ| or $\beta_{i}$ become larger. The sign of the bias is opposite to that of ρ. The right panel shows a graph of the difference in the marginal effect (defined by Equation (11)) as a function of $\alpha_{i}$ and ρ. This graph shows that the absolute value of the difference increases rapidly as $\alpha_{i}$ tends to have a large value, and this difference tends to be larger as the absolute value of ρ becomes larger. Furthermore, the signs of the difference and ρ are different, which is expected for the case where $\gamma_{k}>0.$ These panels imply that an inconsistent nonparametric regression analysis is unavoidable, provided that the GPR model is fitted to the partially observed sample-selection data. Instead, the proposed RSGPR model should be used to correct the sample-selection bias and to estimate the true marginal effect of each predictor in the regression analysis.

## 3. Bayesian Hierarchical Methodology

### 3.1. Hierarchical Representation of the RSGPR Model

Let us revisit the RSGPR model (6) in Section 2.2. From Equations (7) and (8), we see that the log-likelihood function of the RSGPR model based on the partially observed *n*-tuples of observations $\left(y_{i},x_{i},v_{i},s_{i}\right)$ is

(12) $$\begin{array}{ccc} l(\eta_{n_{1}},\gamma ,\rho ,\sigma^{2}) & = & \sum_{i=1}^{n}\left[s_{i}\left\{\ln\bar{F}\left(C_{i};0,1\right)+\ln h\left(y_{i}|s_{i}=1\right)\right\}+\left(1-s_{i}\right)\ln\left\{1-\bar{F}\left(C_{i};0,1\right)\right\}\right]. \end{array}$$

This is a complex function for the Bayesian estimation of the parameters ($\eta_{n_{1}}$ and Ψ) of the RSGPR model. Instead, the following hierarchical representation of the RSGPR model is useful for a simple estimation of the parameters.

First, the likelihood function in Equation (12) can be represented by the following distributional hierarchy.

**Theorem** **1.** *For the n-pairs of independent observations, $(y_{i},s_{i}),$ generated from the RSGPR model defined by Equation (6), their distribution can be written by the following Bayesian hierarchical model:*

$$\begin{array}{ccc} \left[y_{i}|\omega_{i},z_{C_{i}},s_{i}=1\right] & ∼ & N(\eta \left(x_{i}\right)+\zeta z_{C_{i}},\delta \left(\omega_{i}\right)\tau^{2}),\;\;\\ p(s_{i}|z_{i},\omega_{i}) & = & I\left(z_{i}\geq 0\right)I\left(s_{i}=1\right)+I\left(z_{i}<0\right)I\left(s_{i}=0\right),\\ \left[z_{i}|\omega_{i}\right] & ∼ & N(v_{i}^{⊤}\gamma ,\delta \left(\omega_{i}\right)),\\ \omega_{i} & ∼ & G(\omega_{i}),\;\;i=1,\ldots ,n,\\ \eta_{n_{1}} & ∼ & N_{n_{1}}\left(m_{1}\left(x\right),K_{11}\left(x\right)\right),\\ \left[\zeta |\tau^{2}\right] & ∼ & N(\theta_{0},\sigma_{0}\tau^{2}),\\ \tau^{2} & ∼ & \mathrm{IG}(c,d),\\ \gamma & ∼ & N_{q}\left(\gamma_{0},\Omega_{0}\right), \end{array}$$

*where $z_{C_{i}}=z_{i}-v_{i}^{⊤}\gamma ,$ζ=ρσ,$\tau^{2}=\sigma^{2}\left(1-\rho^{2}\right),$IG(c,d) denotes an inverse gamma distribution with the p.d.f. $IG\left(\tau^{2};c,d\right)=d^{c}\tau^{-2(c+1)}e^{-d/\tau^{2}}/\Gamma \left(c\right),$ and G(·) is a distribution function of the scale mixing variable ω.*

When the prior information on ξ, $\tau^{2},$ and γ is not available, a convenient strategy of avoiding improper posterior distribution is to use proper priors with their hyperparameters fixed as appropriate quantity to reflect the diffuseness of the priors (i.e., limiting non-informative priors). For this convenience, the prior distributions in Theorem are used to elicit the prior distributions of ξ, $\tau^{2},$ and γ. All hyperparameters that appeared in the prior distributions of the Bayesian hierarchical model are assumed to be given from the prior information of previous studies or other sources.

### 3.2. Full Conditional Posteriors

Let $y_{n_{1}}={\left(y_{1},\ldots ,y_{n_{1}}\right)}^{⊤}$, $V=(v_{1},\ldots ,v_{n}),$ and $s={\left(s_{1},\ldots ,s_{n}\right)}^{⊤}$ be observed. Further suppose that $z={\left(z_{1},\ldots ,z_{n}\right)}^{⊤}$ and $\omega ={\left(\omega_{1},\ldots ,\omega_{n}\right)}^{⊤}$ are the latent observation vector and the scale mixing vector, respectively. Then, based on the RSGPR model, we obtained joint posterior distribution of $\Theta =\{\eta_{n_{1}},\tau^{2},\zeta ,\gamma ,z,\omega \}$ given the observed data set $D_{n}=\left\{y_{n_{1}},V,s\right\}:$

(13) $$\begin{array}{ccc} p(\Theta \;|\;D_{n}) & \propto & \prod_{i=1}^{n_{1}}\phi \left(y_{i};\eta \left(x_{i}\right)+\zeta z_{C_{i}},\delta \left(\omega_{i}\right)\tau^{2}\right)\\ & \times & \prod_{i=1}^{n}\left[p\left(s_{i}|z_{i},\omega_{i}\right)\phi \left(z_{i};v_{i}^{⊤}\gamma ,\delta \left(\omega_{i}\right)\right)g\left(\omega_{i}\right)\right]\\ & \times & IG\left(\tau^{2};c,d\right)\phi \left(\zeta ;\theta_{0},\sigma_{0}\tau^{2}\right)\phi_{n_{1}}\left(\eta_{n_{1}};m_{1}\left(x\right),K_{11}\left(x\right)\right)\phi_{q}\left(\gamma ;\gamma_{0},\Omega_{0}\right), \end{array}$$

where g(·) is the p.d.f. of the scale mixing variable ω. Note that the joint posterior in (13) is not simplified in an analytic form of the known density and is thus intractable for posterior inference. Instead, we derive conditional posterior distribution of each parameter in Θ in an explicit form, which will be useful for posterior inference by using a Markov chain Monte Carlo (MCMC) method.

Given the joint posterior distribution (13), we can obtain the following posterior distributions whose derivations are provided in Appendix A:

(1) The full conditional posterior distribution of $\eta_{n_{1}}$ is given by
  (14) $$\left[\eta_{n_{1}}\;|\;\Theta_{\setminus \eta_{n_{1}}},D_{n}\right]∼N_{n_{1}}\left(\theta_{\eta_{n_{1}}},\Sigma_{\eta_{n_{1}}}\right),$$
  where $\theta_{\eta_{n_{1}}}=\Sigma_{\eta_{n_{1}}}\left(K_{11}{\left(x\right)}^{-1}m_{1}\left(x\right)+D_{1}{\left(\delta \left(w\right)\right)}^{-1}\left(y_{n_{1}}-\zeta z_{C}\right)/\tau^{2}\right),\;\;\Sigma_{\eta_{n_{1}}}={\left(K_{11}{\left(x\right)}^{-1}+D_{1}{\left(\delta \left(w\right)\right)}^{-1}/\tau^{2}\right)}^{-1},$ $D_{1}\left(\delta \left(\omega \right)\right)=\mathrm{diag}\left\{\delta \left(\omega_{1}\right),\ldots ,\delta \left(\omega_{n_{1}}\right)\right\},$ $z_{C}={\left(z_{C_{1}},\ldots ,z_{C_{n_{1}}}\right)}^{⊤},$ and $z_{C_{i}}=z_{i}-v_{i}^{⊤}\gamma .$
(2) The full conditional posterior distribution of $\tau^{2}$ is an inverse Gamma distribution:
  (15) $$\left[\tau^{2}\;|\;\Theta_{\setminus \tau^{2}},D_{n}\right]∼\mathrm{IG}(c+\frac{n_{1}+1}{2},\;d+\frac{1}{2}\sum_{i=1}^{n_{1}}\frac{{\left(y_{i}-\eta \left(x_{i}\right)-\zeta z_{C_{i}}\right)}^{2}}{\delta (\omega_{i})}+\frac{{\left(\zeta -\theta_{0}\right)}^{2}}{2\sigma_{0}}).$$
(3) The full conditional posterior distribution of ζ is a normal distribution:
  (16) $$\begin{array}{c} \left[\zeta \;|\;\Theta_{\setminus \zeta },D_{n}\right]∼N\left(\theta_{\zeta },\sigma_{\zeta }^{2}\right), \end{array}$$
  where
  $$\theta_{\zeta }=\frac{\theta_{0}/\sigma_{0}+\sum_{i=1}^{n_{1}}\left(y_{i}-\eta \left(x_{i}\right)\right)z_{C_{i}}/\delta \left(\omega_{i}\right)}{1/\sigma_{0}+\sum_{i=1}^{n_{1}}z_{C_{i}}^{2}/\delta \left(\omega_{i}\right)}\;\mathrm{and}\;\sigma_{\zeta }^{2}=(\frac{1}{\sigma_{0}\tau^{2}}+\frac{\sum_{i=1}^{n_{1}}z_{C_{i}}^{2}}{\delta \left(\omega_{i}\right)\tau^{2}}{)}^{-1}.$$
(4) The full conditional posterior distributions of $z_{i}$’s are independent and their distributions are given by
  (17) $$\begin{array}{c} \left[z_{i}|\Theta_{\setminus z_{i}},D_{n}\right]\overset{ind}{∼}\left\{\begin{array}{cc} {\mathrm{TN}}_{\left(-\infty ,0\right)}\left(v_{i}^{⊤}\gamma ,\delta \left(\omega_{i}\right)\right) & \mathrm{if}\;\;s_{i}=0,\\ {\mathrm{TN}}_{\left(0,\infty \right)}\left(\theta_{z_{i}},\sigma_{z_{i}}^{2}\right) & \mathrm{if}\;\;s_{i}=1 \end{array}\right. \end{array}$$
  for i=1,…,n, where
  $$\theta_{z_{i}}=v_{i}^{⊤}\gamma +\frac{\zeta (y_{i}-\eta \left(x_{i}\right))}{\zeta^{2}+\tau^{2}}\;\;\mathrm{and}\;\;\sigma_{z_{i}}^{2}=\frac{\delta \left(\omega_{i}\right)\tau^{2}}{\zeta^{2}+\tau^{2}}.$$
(5) The full conditional posterior density of γ is:
  (18) $$\left[\gamma \;|\;\Theta_{\setminus \gamma },D_{n}\right]\propto N_{q}\left(\theta_{\gamma },\Sigma_{\gamma }\right),$$
  where $\theta_{\gamma }=\Sigma_{\gamma }\left(\sum_{i=1}^{n}\frac{1}{\delta (\omega_{i})}z_{i}v_{i}+\sum_{i=1}^{n_{1}}\frac{\zeta }{\tau^{2}\delta \left(\omega_{i}\right)}\left(\zeta z_{i}+\eta \left(x_{i}\right)-y_{i}\right)v_{i}+\Omega_{0}^{-1}\gamma_{0}\right)$ and $\Sigma_{\gamma }=(\Omega_{0}^{-1}+\sum_{i=1}^{n}\frac{1}{\delta (\omega_{i})}v_{i}v_{i}^{⊤}+\sum_{i=1}^{n_{1}}\frac{\zeta^{2}}{\tau^{2}\delta \left(\omega_{i}\right)}v_{i}v_{i}^{⊤}{)}^{-1}.$
(6) The full conditional posterior densities of $\omega_{i}$’s are independent and they are given by
  (19) $$\begin{array}{ccc} p(\omega_{i}|\Theta_{\setminus \omega_{i}},D_{n}) & \propto & \;\phi \left(y_{i};\eta \left(x_{i}\right)+\zeta z_{C_{i}},\delta \left(\omega_{i}\right)\tau^{2}\right)\phi \left(z_{i};v_{i}^{⊤}\gamma ,\delta \left(\omega_{i}\right)\right)g\left(\omega_{i}\right)I\left(i\leq n_{1}\right)\\ & + & \phi \left(z_{i};v_{i}^{⊤}\gamma ,\delta \left(\omega_{i}\right)\right)g\left(\omega_{i}\right)I\left(i>n_{1}\right). \end{array}$$

### 3.3. Markov Chain Monte Carlo Method

The MCMC scheme, working with the full conditional distributions of the parameters in Θ, is not complicated to implement. A routine Gibbs sampler can be used to generate posterior samples of $\eta_{n_{1}},$ $\tau^{2},$ ζ, $z_{i},$ and γ based on each of their full conditional posterior distributions obtained in Section 3.2. In posterior sampling of $\omega_{i}$’s, Metropolis–Hastings (M–H) within the Gibbs algorithm can be applied because their conditional posterior densities may not have explicit form of known distribution as in Equation (19). For Gibbs sampling, one should note the following points:

(1) Given the initial values of $\Theta^{\left(0\right)},$ the implementation of the Gibbs sampler involves *R* iterative sampling from each of the full conditional posterior distributions obtained in Equation (14) through Equation (19).
(2) Gibbs samples of ρ and $\sigma^{2}$ can be obtained by using those of ζ=ρσ and $\tau^{2}=\sigma^{2}\left(1-\rho^{2}\right)$.
(3) If $\omega_{i}$ degenerates at $\delta (\omega_{i})=1,$ the RSGPR model can be reduced to the SGPR${}_{N}$ model. In this case, the MCMC procedure excludes the Gibbs sampling of $\omega_{i}$’s by using the posterior distribution (19).
(4) For various distributions of mixing variable $\omega_{i}$ and mixing functions $\delta (\omega_{i})$ of the SMN distributions such as $t_{\nu }$, logit, stable, slash, and exponentialpower models (see, e.g., [21,22]).
(5) When $\omega_{i}\overset{iid}{∼}G\left(\nu /2,\nu /2\right)$ and $\delta \left(\omega_{i}\right)=1/\omega_{i},$ the RSGPR model becomes the SGPR${}_{t_{\nu }}$ model. For generating $\omega_{i}$’s, we may use the following posteriors
  (20) $$\begin{array}{ccc} \omega_{i} & \overset{ind}{∼} & \left\{\begin{array}{cc} G\left(\frac{\nu +2}{2},\;\frac{\nu +z_{C_{i}}^{2}}{2}+\frac{{\left(y_{i}-\eta \left(x_{i}\right)-\zeta z_{C_{i}}\right)}^{2}}{2\xi^{2}}\right) & \mathrm{for}\;\;i\leq n_{1},\\ G\left(\frac{\nu +1}{2},\;\frac{\nu +z_{C_{i}}^{2}}{2}\right) & \mathrm{for}\;\;i>n_{1}, \end{array}\right. \end{array}$$
  where $z_{C_{i}}=z_{i}-v_{i}^{⊤}\gamma .$ Except for the SGPR${}_{N}$ and SGPR${}_{t_{\nu }},$ we need to adopt the Metropolis–Hastings algorithm within the Gibbs sampler because the conditional posterior density of $\omega_{i}$ does not have explicit form of known distribution. See [26,27] for the algorithm for sampling $\omega_{i}$ from the posterior density.
(6) When the squared exponential covariance function K(x) in Equation (4) is chosen with unknown hyperparameters $u_{0}$ and $w_{0},$ we need to elicit the priors of $u_{0}$ and $w_{0}$ for the full Bayes methods based on the MCMC method. The priors considered by [28] can be used for this assessment as follows. The prior distributions are a conjugate $u_{0}∼\mathrm{IG}\left(a,b\right)$ and $w_{0}∼\mathrm{HC}\left(c,d\right).$ Here HC(c,d) denotes the half-Cauchy distribution with the p.d.f. $\;HC(w_{0};c,d),$ location parameter c, and scale parameter d. See [28], for compatibility with $w_{0}∼\mathrm{HC}\left(c,d\right)$ to elicit the prior information on $w_{0}.$
(7) Full conditional posterior distributions of $u_{0}$ and $w_{0}$ are
  $$\left[u_{0}|\Theta ,w_{0},D\right]∼\mathrm{IG}\left(a^{*},b^{*}\right)\;\;\mathrm{and}\;\;p\left(w_{0}|\Theta ,u_{0},D\right)\propto \phi_{n_{1}}\left(\eta_{n_{1}};m_{1}\left(x\right),K_{11}\left(x\right)\right)HC\left(w_{0};c,d\right),$$
  where $a^{*}=a+n_{1}/2$ and $b^{*}=b+u_{0}{\left(\eta_{n_{1}}-m_{1}\left(x\right)\right)}^{⊤}K_{11}{\left(x\right)}^{-1}\left(\eta_{n_{1}}-m_{1}\left(x\right)\right).$ Note that the conditional posterior density of $w_{0}$ does not have explicit form of known distribution. This implies the use of the Metropolis–Hastings algorithm within the Gibbs sampler to generate $w_{0}$ from the posterior density.
(8) After obtaining the Gibbs samples of Θ, we can use them for Monte Carlo estimation of regression function $\eta_{n_{2}}$ and missing observations $y_{n_{2}}.$ They can be also used for predicting regression functions and $y_{i}^{\prime }$s evaluated at new predictors (see, e.g., [26]).

### 3.4. Prediction with Bias Corrected Regression Function

According to the Gaussian (i.e., MaxEnt) process prior, the joint distribution of the training outputs ($\eta_{n_{1}}$) and test outputs $\left(\eta_{n_{2}}\right)$ is

$$\left(\begin{array}{c} \eta_{n_{1}}\\ \eta_{n_{2}} \end{array}|\;x\right)∼N_{n}\left(m\left(x\right)=\left(\begin{array}{c} m_{1}\left(x\right)\\ m_{2}\left(x\right) \end{array}\right),K\left(x\right)=[\begin{array}{cc} K_{11}\left(x\right) & K_{12}\left(x\right)\\ K_{21}\left(x\right) & K_{22}\left(x\right) \end{array}]\right),$$

where $\eta_{n}={\left(\eta_{n_{1}}^{⊤},\eta_{n_{2}}^{⊤}\right)}^{⊤},$ $K_{12}\left(x\right)$ denotes the $n_{1}\times n_{2}$ matrix of the covariances evaluated at all pairs of training points $\left\{x_{i}|i=1,\ldots ,n_{1}\right\}$ and test points $\{x_{j}|j=n_{1}+1,\ldots ,n\},$ and similarly for the other entities $K_{11}\left(x\right),$ $K_{21}\left(x\right),$ $K_{22}\left(x\right).$ The RSGPR framework provides a straightforward way of predicting test outputs based on the relevant test points and the training outputs. Conditioning the joint Gaussian prior distribution on the training observations, we arrive at the predictive distribution for the future (or missing) regression function given by

(21) $$\left[\eta_{n_{2}}|\eta_{n_{1}},x\right]∼N_{n_{2}}\left(m_{2}\left(x\right)+K_{21}\left(x\right)K_{11}{\left(x\right)}^{-1}\left(\eta_{n_{1}}-m_{1}\left(x\right)\right),K_{22}\left(x\right)-K_{21}\left(x\right)K_{11}{\left(x\right)}^{-1}K_{12}\left(x\right)\right).$$

The regression function ($\eta_{n_{2}}$) value can be sampled from the predictive distribution (21) by evaluating the mean and covariance matrix of the distribution. Thus, it can be generated within the preceding MCMC algorithm for estimating the RSGPR model: We can generate $\eta_{n_{2}}$ and unobserved observation vector $y_{n_{2}}={\left(y_{N_{1}+1},\ldots ,y_{n}\right)}^{⊤}$ in the *r*-th iteration of the algorithm whose Markov chain is augmented by the following conditional distributions.

$$\begin{array}{ccc} \left[\eta_{n_{2}}^{\left(r\right)}|\eta_{n_{1}}^{\left(r\right)},x\right] & ∼ & N_{n_{2}}\left(m_{2}\left(x\right)+K_{21}\left(x\right)K_{11}{\left(x\right)}^{-1}\left(\eta_{n_{1}}^{\left(r\right)}-m_{1}\left(x\right)\right),K_{22}\left(x\right)-K_{21}\left(x\right)K_{11}{\left(x\right)}^{-1}K_{12}\left(x\right)\right),\\ \left[y_{n_{2}}^{\left(r\right)}|\Theta^{\left(r\right)},y_{n_{1}},x\right] & ∼ & N(\eta_{n_{2}}^{\left(r\right)},\;\sigma^{2,(r)}D_{2}{\left(\delta \left(\omega \right)\right)}^{\left(r\right)}), \end{array}$$

where $\eta_{n_{2}}^{\left(r\right)}={\left(\eta {\left(x_{n_{1}+1}\right)}^{\left(r\right)},\ldots ,\eta {\left(x_{n}\right)}^{\left(r\right)}\right)}^{⊤}$ and $D_{2}{\left(\delta \left(\omega \right)\right)}^{\left(r\right)}=\mathrm{diag}\left\{\delta {\left(\omega_{n_{1}+1}\right)}^{\left(r\right)},\ldots ,\delta {\left(\omega_{n}\right)}^{\left(r\right)}\right\}.$ Let $\eta_{n_{2}}^{\left(1\right)},\ldots ,\eta_{n_{2}}^{\left(R\right)}$ and $y_{n_{2}}^{\left(1\right)},\ldots ,y_{n_{2}}^{\left(R\right)}$ are respective samples generated from *R* iterations, then bias corrected expected value of $\eta_{n_{2}}$ and that of posterior predictive distribution of $y_{n_{2}}$ can be approximated via Monte Carlo by

$${\hat{\eta }}_{n_{2}}=E\left[\eta_{n_{2}}|x\right]\approx \frac{1}{R}\sum_{r=1}^{R}\eta_{n_{2}}^{\left(r\right)}\;\;\mathrm{and}\;\;\;E\left[y_{n_{2}}|y_{n_{1}},x\right]\approx \frac{1}{R}\sum_{r=1}^{R}y_{n_{2}}^{\left(r\right)}.$$

Note that $\mathrm{Cov}\left(\eta_{n_{2}}|x\right)=K_{22}\left(x\right)-K_{21}\left(x\right)K_{11}{\left(x\right)}^{-1}K_{12}\left(x\right).$

## 4. Numerical Illustrations

This section presents empirical results of the Bayesian hierarchical RSGPR analysis of non-normal data with the sample selection. We provide results obtained from simulated data applications comparing the performance of the RSGPR model with that of the GPR model. We developed our program written in R (see, e.g., [29]), which is available from the authors upon request.

### 4.1. Simulation Scheme

In this simulation, we evaluated the finite-sample performance of the RSGPR model by using sample-selection data generated for different sizes. The performance was assessed in terms of sample-selection bias correction and robustness to non-normal model errors. These could be measured by comparing the posterior estimation and prediction results of the RSGPR model with those based on the GPR model. Specifically, we compared the results obtained from the SGPR${}_{N}$ (or SGPR${}_{t_{10}}$) analysis with the results of the GPR (or GPR${}_{t_{10}}$) analysis based on a partially observed sample-selection data. This study also demonstrated that the SGPR${}_{t_{\nu }}$ model is more robust against outliers compared to the SGPR${}_{N}$ model. To evaluate the performance, we generated M=300 sets of partially observed sample-selection data with size n=300 with $n_{1}=150$ (i.e., the missing rate is 0.5) from each of the three models (see details below). The general form of the three models is as follows:

(22) $$\begin{array}{ccc} y_{i} & = & \;\left\{\begin{array}{cc} 50\;x_{i}+5\;\sin\left(10x_{i}\right)+\epsilon_{i} & \mathrm{for}\;\;s_{i}=1,\;\;x_{i}\in \left(0,1\right),\\ \mathrm{missing} & \mathrm{for}\;\;s_{i}=0,\;\;i=1,\ldots ,n, \end{array}\right.\\ z_{i} & = & \gamma +\varepsilon_{i},\;\;\;\left(\begin{array}{c} \epsilon_{i}\\ \varepsilon_{i} \end{array}\right)\overset{iid}{∼}{\mathrm{SMN}}_{2}\left(0,\Sigma ,\delta ,G\right),\; \end{array}$$

where $s_{i}=I\left(z_{i}\geq 0\right),$ γ=0, ρ=0.5, and σ=3.

Model 1 was defined by assuming that the distribution G degenerates at ω=1. Model 2 was obtained from the model (22) by setting δ(ω)=1/ω and G∼G(10/2,10/2). Model 3 assumed a mixture of bivariate normal errors instead of the ${\mathrm{SMN}}_{2}\left(0,\Sigma ,\delta ,G\right)$ distribution. Throughout our simulation, the hyper-parameters for the Bayesian hierarchical model in Theorem 1 were chosen to reflect the diffuseness of the priors. To obtain the limiting non-informative priors of ζ, $\tau^{2}$ and γ, their hyper-parameters were assessed as $\theta_{0}=0,$ $\sigma_{0}=10,$ c=0.001, d=0.001, $\gamma_{0}=0,$ and $\Omega_{0}=10.$ Note that when our observational data were augmented through proper prior information, as in this simulation study, the issue to identify the parameters in the RSGPR model disappeared.

In the simulation, we proceeded as follows to estimate the parameters. Using each of M=300 datasets generated from the models (Model 1, Model 2, and Model 3), we fitted the RSGPR and GPR models and applied the proposed Bayesian hierarchical methodology to estimate the parameters of the fitted models by assuming the above prior distributions. To implement the methodology by using each generated dataset, we obtained 15,000 posterior samples from the developed MCMC algorithm (in Section 3) with 5 thinning periods after a burn-in period of 5000 samples. This sampling plan guaranteed a convergence of the chain of the MCMC algorithm. The MCMC method (applied to each of M=300 datasets) gave estimates (or predictions) of the nonparametric regression function (η(x)) as well as the other parameters of the RSGPR model.

**Figure 2:**
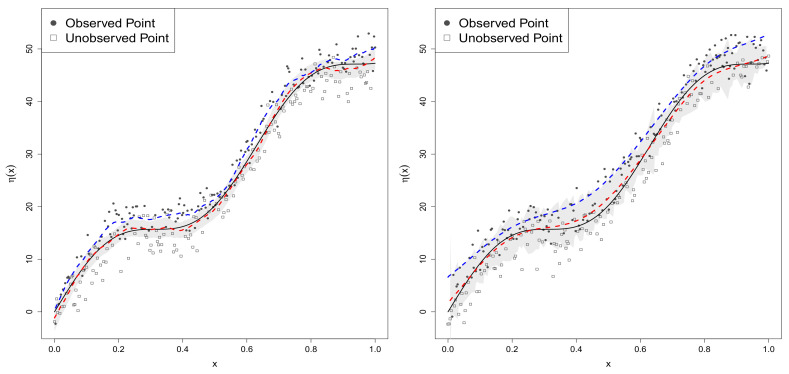
Graphs of estimated regression functions (**left panel**) and predicted regression functions (**right panel**): (**i**) black lines are used for the true regression function; (**ii**) red dashed lines for the robust sample-selection Gaussian process regression (RSGPR) models; (**iii**) blue dashed lines for the Gaussian process regression (GPR) models.

**Figure 3:**
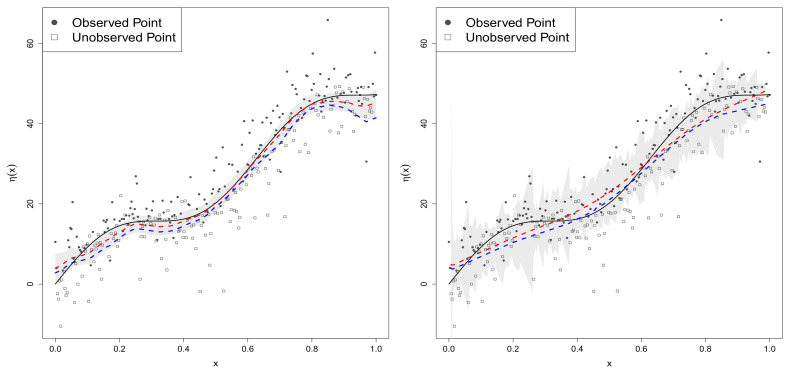
Graphs of regression functions: estimated regression functions (**left panel**) and predicted regression functions (**right panel**).

The variability in the regression function estimates (${\hat{\eta }}_{n_{1}}$) and predictions (${\hat{\eta }}_{n_{2}}$) obtained by using a dataset were then visualized as shown in Figure 2 and Figure 3. These figures compare the estimates (or predictions) of the nonparametric regression function obtained from two models (the RSGPR and GPR models). The black line of each graph in the figures shows the true regression function of the model (22). The red dashed line denotes the posterior mean (or predicted value) of the regression function of the model (22) obtained by using a Bayesian hierarchical RSGPR analysis with the sample-selection data of size $n_{1}=150,$ while the blue dashed line depicts that obtained by using a GPR analysis of the sample-selection data. The 97.5th quantile and 2.5th quantile of 3000 posterior samples (predictions) of each regression function ($\eta (x_{i})$) in the RSGPR model were also calculated. In each figure, these quantiles were used to draw 95% posterior (or prediction) intervals of $\eta (x_{i})$’s by using the gray band. The accuracy of parameter estimates was calculated by using the mean absolute bias (MAB) and the root mean square error (RMSE):

$$\mathrm{MAB}\;=\;\frac{1}{M}\sum_{k=1}^{M}\sum_{\ell =1}^{p}|{\hat{\theta }}_{k\ell }-\theta_{\ell }|\;\mathrm{and}\;\mathrm{RMSE}={\left\{\frac{1}{M}\sum_{k=1}^{M}\sum_{\ell =1}^{p}{\left({\hat{\theta }}_{k\ell }-\theta_{\ell }\right)}^{2}\right\}}^{1/2},$$

where M=300 and ${\hat{\theta }}_{k\ell }$ is the posterior estimate of *ℓ*-th element of p×1 parameter vector θ in the *k*-th replication.

### 4.2. Performance of the RSGPR Model

#### 4.2.1. Sample-Selection Data Generated from Model 1

If the distribution *G* is degenerated at ω=1, we then obtain the SGPR${}_{N}$ model from the RSGPR model (6). Using each of M=300 datasets generated from Model 1, the proposed Bayesian hierarchical methodology was applied to estimate the parameters of the model. If we set ρ=0, the methodology yielded posterior estimates of the GPR model. The estimation results for the parameter $\eta_{n_{1}}$ of our primary interest, based on the SGPR${}_{N}$ and GPR models, are shown in the left panel of Figure 2. The left panel provides the following results. First, the posterior estimates of $\eta_{n_{1}}$ based on the proposed SGPR${}_{N}$ model (red dashed line) are close to their true values (black line), while those based on the GPR model (blue dashed line) tend to have severe sample-selection bias. Second, when the SGPR${}_{N}$ model was used to fit the generated sample-selection dataset, the posterior estimates of regression function based on the model concentrated true values of the $\eta (x_{i})$’s as shown in their 95% posterior intervals (gray band). Third, the difference between the true regression function (black line) and the estimated regression function obtained by using the GPR model (the blue dashed line) confirms Lemma 4, which shows the existence of the sample-selection bias in the GPR regression for data with the sample selection. In summary, the left panel of Figure 2 illustrates the existence of the sample-selection bias in the GPR analysis with the sample-selection data, as discussed in Section 2.3. It also demonstrates the performance of the proposed methodology based on the SGPR${}_{N}$ model to eliminate the sample-selection bias (or inconsistency in estimating the regression function), which could not be achieved by using the GPR model.

The mean of M=300 estimation results for the other parameters were listed in Table 1. As shown in Table 1, the MCMC parameter estimates were close to their true values for the SGPR${}_{N}$ model, while those based on the GPR model were severely biased. In addition, the MAB and RMSE values in the table ensure that the performance of the SGPR${}_{N}$ model is far better than the GPR model when the sample selection data was used for Bayesian nonparametric regression analysis. For both models, the small values of Monte Carlo (MC) error (compared to the RMSE) of each parameter suggests that approximate convergence was reached and the sequence generated from the MCMC samples was well mixed.

#### 4.2.2. Data Generated from Model 2

The proposed Bayesian hierarchical methodology for the SGPR${}_{t_{10}}$ model was applied to each of M=300 datasets generated from Model 2. The SGPR${}_{t_{10}}$ model can be obtained from the SGPR model (6) by setting δ(ω)=1/ω and ω∼G(10/2,10/2). If we set ρ=0, the methodology could also be used to obtain posterior samples to estimate the GPR${}_{t_{10}}$ model (GPR model with $t_{10}$ errors). The results of the simulation appear in the right panel of Figure 2 and Table 1. Graphs in the right panel of Figure 2 depict the prediction results of $\eta_{n_{2}}$ based on the SGPR${}_{t_{10}}$ and GPR${}_{t_{10}}$ models. The graphs clearly show that the sample-selection bias in predicting $\eta (x_{i})$’s based on the GPR${}_{t_{10}}$ model is too large to allow for a prediction of the true regression function $\eta_{n_{2}}$ (or future regression function). However, the proposed methodology using the SGPR${}_{t_{10}}$ model correctly predicted the true regression function; see 95% prediction interval and ${\hat{\eta }}_{n_{2}},$ i.e., red dashed line. The prediction of $\eta_{n_{2}}$ based on the SGPR${}_{t_{10}}$ model is far better than that based on the GPR${}_{t_{10}}$ model. Compared to the GPR${}_{t_{10}}$ model, the methodology based on the SGPR${}_{t_{10}}$ model yields smaller MAB and smaller RMSE of the parameter estimates; see Table 1. Table 1 shows that the parameter estimates of the SGPR${}_{t_{10}}$ model with heavy-tailed errors tend to produce larger estimation errors (MAB and RMSE) than those of the SGPR${}_{N}$ model. The results of the above simulation demonstrate the superior performance of the SGPR${}_{t_{10}}$ model and the usefulness of the proposed Bayesian hierarchical methodology to remedy the sample-selection bias in the prediction that occurred in the GPR${}_{t_{10}}$ analysis of the sample-selection data.

#### 4.2.3. Data Generated from Model 3 with Normal Mixture Errors

We generated datasets from Model 3 with size n=300. Model 3 was defined by the model (22) with independent bivariate normal mixture errors: viz.

$$0.4\;N_{2}\left(0,\Sigma_{\left(1\right)}\right)+0.2\;N_{2}\left(0,\Sigma_{\left(2\right)}\right)+0.2\;N_{2}\left(0,\Sigma_{\left(4\right)}\right)+0.1\;N_{2}\left(0,\Sigma_{\left(8\right)}\right)+0.1\;N_{2}\left(0,\Sigma_{\left(16\right)}\right),$$

where 50% of the outcomes were missing in each dataset and $\Sigma_{\left(k\right)}$ was equal to Σ whose value of $\sigma^{2}$ was 9k. The generated dataset was fitted to the SGPR${}_{N}$, SGPR${}_{t_{5}}$, and SGPR${}_{t_{10}}$ models in turn. Based on posterior samples, we calculated the Bayes estimates of the three models’ parameters together with their deviance information criterion (DIC) values introduced by [30]. The average DIC values obtained from the dataset were found to be 2727.06, 1477.43, 1396.24 for the SGPR${}_{N}$, SGPR${}_{t_{10}}$, and SGPR${}_{t_{5}}$ models, respectively. This suggested that the SGPR${}_{t_{5}}$ model is the best fitting model among the three models, while the SGPR${}_{N}$ model is the worst.

The graphs in the left panel of Figure 3 show the true regression function (black line) and estimated regression functions (${\hat{\eta }}_{n_{1}}$) under the best fitting SGPR${}_{t_{5}}$ model (red line) and the SGPR${}_{N}$ model (blue line). The graphs in the right panel of Figure 3 depict predicted regression function (${\hat{\eta }}_{n_{2}}$) based on the best fitted model (in red), the SGPR${}_{N}$ model (in blue), and the true regression function (in black). Even though the best fitted model based on bivariate $t_{5}$ error distributions was misspecified, 95% posterior intervals (or prediction intervals) of $\eta (x_{i})$ obtained from the SGPR${}_{t_{5}}$ model did include the true regression function values (see gray bands in Figure 3). The prediction result of the SGPR${}_{t_{5}}$ and SGPR${}_{N}$ models are very wild due to outliers generated by the normal mixture errors, while the graphs of the SGPR${}_{t_{5}}$ are more robust for the model misspecification.

## 5. Conclusions

This study considered a MaxEnt approach to develop a Bayesian nonparametric regression analysis of non-normal data with the sample selection. For this purpose, by using Boltzmann’s maximum entropy theorem, we introduced a MaxEnt process regression model that reflects partial prior information for an uncertain regression function. We found that a special case of the MaxEnt regression model reduced to the well-known GPR model. Second, we generalized the GPR model to propose the RSGPR model and explored its theoretical properties. These properties showed that the new model was well-designed to correct the sample-selection bias and implement a robust GPR analysis. Third, we developed a hierarchical RSGPR model based on a stochastic representation of the RSGPR model and proposed a Bayesian hierarchical methodology for the RSGPR analysis of a non-normal data with sample selection. A simulation study showed that the finite sample performance of the proposed methodology eliminated the sample selection bias and estimated the population model parameters with robustness and high accuracy. In a comparative numerical study on the analysis of nonparametric regression models with sample selection data, we found that the estimation results using the RSGPR model outperformed those using the GPR model for both in-sample estimation and out-of-sample forecasts.

The theoretical results of the RSGPR model and the methodology for the RSGPR analysis proposed in this study have several interesting issues that are worth considering further. First, the RSGPR framework using the MaxEnt process prior can be generalized to the so called *stochastically constrained RSGPR regression* that uses the constrained MaxEnt process as the prior distribution of the regression function with uncertain constraints. Second, an empirical study with real data as well as an asymptotic evaluation, such as consistency, would be particularly noteworthy to explore. For example, estimating monotone regression function with or without uncertainty and testing the monotonicity of the regression function can be considered in the context of a constrained RSGPR analysis with sample-selection data. Finally, the Bayesian hierarchical methodology can be broadened in various regression models with the general class of skew-SMN error distributions considered by [11]. For example, this methodology can be applied to a von Bertalanffy growth curve analysis of heavy-tailed fishery data with sample selection (see, e.g., [28]). We hope to address all of these in the future.

## Figures and Tables

**Table 1:** Posterior Summary.

True Value	Mean	s.d.	SGPR${}_{N}$ Model		MC Error	Mean	s.d.	GPR Model		MC Error
			RMSE	MAB				RMSE	MAB	
σ=3	2.831	0.308	0.351	0.426	0.018	2.094	0.104	0.912	0.800	0.002
ρ=0.5	0.380	0.376	0.563	0.287	0.064	NA	NA	NA	NA	NA
			**SGPR** ${}_{t_{10}}$ **Model**					**GPR** ${}_{t_{10}}$ **Model**		
σ=3	2.880	0.974	0.509	0.515	0.050	2.130	0.109	0.876	0.800	0.003
ρ=0.5	0.435	0.275	0.627	0.422	0.032	NA	NA	NA	NA	NA

s.d.: standard deviation; SGPR${}_{N}$: sample-selection Gaussian process normal error regression; RMSE: root mean square error; MAB: mean absolute bias; MC: Monte Carlo; GPR: Gaussian process regression.

## Appendix A

### Appendix A.1. Proof of Lemma 1

**Proof.** The proof proceeds along the lines of Corollary 1 of [18] by changing the partial prior information on θ to that on $\eta_{n}\left(x\right).$ ☐

### Appendix A.2. Proof of Lemma 2

**Proof.** Equation (8) shows that the distribution of $\left[y_{i}|\eta \left(x_{i}\right),s_{i}=1\right]$ is skew-${\mathrm{SMN}}_{p}\left(C_{i};\theta_{i},\Sigma ,\kappa ,G\right)$ with the density (8). Thus, the result by [13] yields a stochastic representation of a conditional skew-${\mathrm{SMN}}_{p}\left(C_{i};\theta ,\Sigma ,\kappa ,G\right)$ distribution given on ω, which is

$$\begin{array}{ccc} \left[y_{i}|\omega ,\eta \left(x_{i}\right),s_{i}=1\right] & \overset{d}{=} & \eta \left(x_{i}\right)+\rho \sigma Z_{C_{i}}+\sigma {\left(1-\rho^{2}\right)}^{1/2}U_{i}, \end{array}$$

where $U_{i}$ is independent of $Z_{C_{i}}.$ Introducing ω∼G(ω) to the conditional stochastic representation, we have the two-stages of distributional hierarchy for the distribution of $[y_{i}|\eta \left(x_{i}\right)s_{i}=1].$ ☐

### Appendix A.3. Proof of Lemma 3

**Proof.** For the RSGPR model, let $z_{1}={\left(z_{1},\ldots ,z_{n_{1}}\right)}^{⊤}$ be the latent variables vector which corresponds to the observed vector $y_{n_{1}}.$ Then, for fixed $\eta_{n_{1}}$ and ω, the joint distribution of $y_{n_{1}}$ and $z_{1}$ is ${\left(y_{n_{1}}^{⊤},z_{1}^{⊤}\right)}^{⊤}∼N_{2n_{1}}\left(\mu^{*},\delta \left(\omega \right)\Sigma ⊗I_{n_{1}}\right),$ where $\mu^{*}={\left(\eta_{n_{1}}^{⊤},\mu_{1}^{⊤}\right)}^{⊤}$ and $\mu_{1}={\left(v_{1}^{⊤}\gamma ,\ldots ,v_{n_{1}}^{⊤}\gamma \right)}^{⊤}.$ This yields the density of selected observations (i.e., $\left[y_{n_{1}}|{\left(\delta \left(\omega \right)\right)}^{-1/2}\left(z_{1}-\mu_{1}\right)\in C_{\alpha }\right]$ is given by

$$f\left(y_{n_{1}}|\eta_{n_{1}},\Psi \right)=\left[\phi_{n_{1}}\left(y_{1};\eta_{n_{1}},\delta \left(\omega \right)\sigma^{2}I_{n_{1}}\right){\bar{\Phi }}_{n_{1}}\left(C_{\alpha };\frac{\rho }{\sigma }\left(y_{n_{1}}-\eta_{n_{1}}\right),\left(1-\rho^{2}\right)I_{n_{1}}\right)\right]/{\bar{\Phi }}_{n_{1}}\left(C_{\alpha };0,I_{n_{1}}\right),$$

where $\phi_{p}\left(\cdot ;\mu ,\Sigma \right)$ is the *p*-variate normal density with mean vector μ and covariance matrix Σ, and ${\bar{\Phi }}_{p}\left(C;\mu ,\Sigma \right)=\int_{C}\phi_{p}\left(v;\mu ,\Sigma \right)dv.$ Applying the Gaussian process prior $p_{0}\left(\eta_{n_{1}}\right)\propto \exp\left\{-\frac{1}{2}({\left(\eta_{n_{1}}-m_{1}\left(x\right)\right)}^{⊤}K_{11}{\left(x\right)}^{-1}\left(\eta_{n_{1}}-m_{1}\left(x\right)\right)\right\}$ to the likelihood yields a conditional posterior density:

$$\begin{array}{c} p\left(\eta_{n_{1}}|y_{n_{1}},\Psi \right)=\left[\phi_{n_{1}}\left(\eta_{n_{1}};\theta_{1},\Omega_{1}\right){\bar{\Phi }}_{n_{1}}\left(C_{\alpha };\theta_{2}+\Gamma \Omega^{-1}\left(\eta_{n_{1}}-\theta_{1}\right),\Omega_{2}-\Gamma \Omega_{1}^{-1}\Gamma^{⊤}\right)\right]/{\bar{\Phi }}_{n_{1}}\left(C_{\alpha };\theta_{2},\Omega_{2}\right), \end{array}$$

which is the skew-normal distribution whose properties were well developed by [13,23]. According to this literature, we can easily obtain the stochastic representation (10). ☐

### Appendix A.4. Proof of Corollary 2

**Proof.** Setting η(ω)=1 (i.e., the distribution of ω degenerates at ω=1 and η(ω)=ω, the RSGPR model reduces to the SGPR${}_{N}$ model. Applying Lemma 3 for the SGPR${}_{N}$ model and the mean of a truncated normal distribution given in [31] yield the conditional posterior mean of the regression function: $\;E\left[\eta_{n_{1}}|y_{n_{1}},\Psi \right]=\theta_{1}+\Gamma \Omega_{2}^{-1}E\left[W_{1}^{C_{\beta }}\right].$ Difference between this posterior mean with that in the first $n_{1}\times 1$ sub-vector of the Equation (5) gives the result. ☐

### Appendix A.5. Proof of Corollary 3

**Proof.** Under the RSGPR model $E\left[y_{i}|\eta \left(x_{i}\right),s_{i}=1\right]=\eta \left(x_{i}\right)+\rho \sigma E\left[\delta_{1}\left(v_{i}^{⊤}\gamma ,\omega \right)\right]$ by Lemma 2 and the expression (see, e.g., [31]) of $E[Z_{C_{i}}|\omega ],$ where $\eta (x_{i})$ is the regression function and $\alpha_{i}=-v_{i}^{⊤}\gamma .$ A straightforward derivation of $E[y_{i}|\eta \left(x_{i}\right),s_{i}=1]$ with respect to $x_{ki}$ gives

$$\frac{\partial E[y_{i}|\eta \left(x_{i}\right),s_{i}=1]}{\partial x_{ki}}=\;\frac{\partial \eta (x_{i})}{\partial x_{ki}}+\gamma_{k}\rho \sigma E_{\omega }\left[\frac{1}{\delta (\omega )}\left(\delta_{2}\left(v_{i}^{⊤}\gamma ,\omega \right)-\delta_{1}{\left(v_{i}^{⊤}\gamma ,\omega \right)}^{2}\right)\right].$$

Comparing with $\frac{\partial E[y_{i}|\eta \left(x_{i}\right)]}{\partial x_{ki}}=\frac{\partial \eta (x_{i})}{\partial x_{ki}}$ for the GPR model, we see that the expression (11) is the magnitude of the sample-selection bias in estimating the marginal effect of the *k*-th independent variable. ☐

### Appendix A.6. Proof of Theorem 1

**Proof.** The first four stages of the distributional hierarchy reduce to the stochastic representation in Lemma 2 where marginal density of $\left[y_{i}|s_{i}=1\right]$ is $h(y_{i})$ which is given by Equation (8). We also see that the 2nd to 4th stages of the distributional hierarchy yield the probability mass function $p(s_{i}),$ which is

$$\int_{0}^{\infty }\int_{-\infty }^{\infty }p\left(s_{i}|z_{i},\omega_{i}\right)\phi \left(z_{i};v_{i}^{⊤}\gamma ,\delta \left(\omega_{i}\right)\right)dz_{i}dG\left(\omega_{i}\right)=\bar{F}{\left(C_{i};0,1\right)}^{s_{i}}{\left(1-\bar{F}\left(C_{i};0,1\right)\right)}^{1-s_{i}}.$$

This is equivalent to Equation (7). As a result, the logarithm of the joint density of the *n* pairs of independent observations, $\left(y_{i},s_{i}\right)$ under the hierarchy is equal to the right hand side of Equation (12). Thus, the first four stages of the hierarchy defines a hierarchical RSGPR model. Introducing the GP prior of $\eta_{n_{1}}$ and priors of Ψ to elicit our prior information about them, we have the Bayesian hierarchical model. ☐

### Appendix A.7. Derivation of Conditional Posterior Distributions

(1) Full conditional distribution of $\eta_{n_{1}}$: Equation (13) states that the full conditional density of $\eta_{n_{1}}$ is
  $$\begin{array}{ccc} p\left(\eta_{n_{1}}\;|\;\Theta_{\setminus \eta_{n_{1}}},D_{n}\right) & \propto & \phi_{n_{1}}\left(y_{n_{1}};\eta_{n_{1}}+\zeta z_{C},\tau^{2}D_{1}\left(\delta \left(w\right)\right)\right)\phi_{n_{1}}\left(\eta_{n_{1}};m_{1}\left(x\right),K_{11}\left(x\right)\right),\\ & \propto & \exp\left\{-\frac{1}{2}\eta_{n_{1}}^{⊤}\Sigma_{\eta_{n_{1}}}^{-1}\eta_{n_{1}}+\theta_{\eta_{n_{1}}}^{⊤}\Sigma_{\eta_{n_{1}}}^{-1}\eta_{n_{1}}\right\},\\ & \propto & \exp\left\{-\frac{1}{2}{\left(\eta_{n_{1}}-\theta_{\eta_{n_{1}}}\right)}^{⊤}\Sigma_{\eta_{n_{1}}}^{-1}\left(\eta_{n_{1}}-\theta_{\eta_{n_{1}}}\right)\right\}, \end{array}$$
  which is the kernel of $N_{n_{1}}\left(\theta_{\eta_{n_{1}}},\Sigma_{\eta_{n_{1}}}\right)$ distribution.
(2) Full conditional distribution of $\tau^{2}$: We see from Equation (13) that the full conditional posterior density is
  $$\begin{array}{ccc} p(\tau^{2}|\Theta_{\setminus \tau^{2}},D_{n}) & \propto & \prod_{i=1}^{n_{1}}\phi \left(y_{i};\eta \left(x_{i}\right)+\zeta z_{C_{i}},\delta \left(\omega_{i}\right)\tau^{2}\right)IG\left(\tau^{2};c,d\right)\phi \left(\zeta ;\theta_{0},\sigma_{0}\tau^{2}\right),\\ & \propto & \tau^{-(n_{1}+2c+3)}\exp\left\{-\left(d+\frac{1}{2}\sum_{i=1}^{n_{1}}\frac{{\left(y_{i}-\eta \left(x_{i}\right)-\zeta z_{C_{i}}\right)}^{2}}{\delta (\omega_{i})}+\frac{{\left(\zeta -\theta_{0}\right)}^{2}}{2\sigma_{0}}\right)/\tau^{2}\right\}. \end{array}$$
  This is the kernel of $\mathrm{IG}\left(c+\frac{n_{1}+1}{2},\;d+\frac{1}{2}\sum_{i=1}^{n_{1}}\frac{{\left(y_{i}-\eta \left(x_{i}\right)-\zeta z_{C_{i}}\right)}^{2}}{\delta (\omega_{i})}+\frac{{\left(\zeta -\theta_{0}\right)}^{2}}{2\sigma_{0}}\right)$ distribution.
(3) Full conditional distribution of ζ: Equation (13) gives the full conditional density of ζ given by
  $$\begin{array}{ccc} p(\zeta |\Theta_{\setminus \zeta },D_{n}) & \propto & \prod_{i=1}^{n_{1}}\phi \left(y_{i};\eta \left(x_{i}\right)+\zeta z_{C_{i}},\delta \left(\omega_{i}\right)\tau^{2}\right)\phi \left(\zeta ;\theta_{0},\sigma_{0}\tau^{2}\right),\\ & \propto & \exp\left\{-\frac{\zeta^{2}-2\theta_{\zeta }\zeta }{2\sigma_{\zeta }^{2}}\right\}\propto \exp\left\{-\frac{{\left(\zeta -\theta_{\zeta }\right)}^{2}}{2\sigma_{\zeta }^{2}}\right\}, \end{array}$$
  which is the kernel of $N(\theta_{\zeta },\sigma_{\zeta }^{2})$ distribution.
(4) Full conditional distributions of $z_{i}$’s: Equation (13) indicates that the full conditional posterior densities of $z_{i}$s are mutually independent, and that for each *i*,
  $$\begin{array}{ccc} p(z_{i}|\Theta_{\setminus_{i}},D_{n}) & \propto & {\left[\phi \left(y_{i};\eta \left(x_{i}\right)+\zeta \left(z_{i}-v_{i}^{⊤}\gamma \right),\delta \left(\omega_{i}\right)\tau^{2}\right)\phi \left(z_{i};v_{i}^{⊤}\gamma ,\delta \left(\omega_{i}\right)\right)\right]}^{s_{i}}{\left[\phi (z_{i};v_{i}^{⊤}\gamma ,\delta \left(\omega_{i}\right))\right]}^{1-s_{i}}\\ & \propto & [\phi (z_{i};\theta_{z_{i}},\sigma_{z_{i}}^{2}{)]}^{s_{i}}{\left[\phi (z_{i};v_{i}^{⊤}\gamma ,\delta \left(\omega_{i}\right))\right]}^{1-s_{i}}. \end{array}$$
  Since the support of $z_{i}$ is $\left\{z_{i};z_{i}\geq 0\right\}$ for $s_{i}=1,$ while $\left\{z_{i};z_{i}<0\right\}$ for $s_{i}=0,$ we have the truncated normal distributions.
(5) Full conditional distribution of γ: The full conditional posterior density of γ is given by
  $$\begin{array}{ccc} p(\gamma |\Theta_{\setminus \gamma },D_{n}) & \propto & \prod_{i=1}^{n_{1}}\phi \left(y_{i};\eta \left(x_{i}\right)+\zeta \left(z_{i}-v_{i}^{⊤}\gamma \right),\delta \left(\omega_{i}\right)\tau^{2}\right)\\ & \times & \phi_{q}\left(\gamma ;\gamma_{0},\Omega_{0}\right)\prod_{i=1}^{n}\phi \left(z_{i};v_{i}^{⊤}\gamma ,\delta \left(\omega_{i}\right)\right)\\ & \propto & \exp\left\{-\frac{1}{2}{\left(\gamma -\theta_{\gamma }\right)}^{⊤}\Sigma_{\gamma }^{-1}\left(\gamma -\theta_{\gamma }\right)\right\}, \end{array}$$
  which is the kernel of $N_{q}\left(\theta_{\gamma },\Sigma_{\gamma }\right)$ distribution.
